# Latency-Associated Expression of Human Cytomegalovirus US28 Attenuates Cell Signaling Pathways To Maintain Latent Infection

**DOI:** 10.1128/mBio.01754-17

**Published:** 2017-12-05

**Authors:** Benjamin A. Krishna, Emma L. Poole, Sarah E. Jackson, Martine J. Smit, Mark R. Wills, John H. Sinclair

**Affiliations:** a Department of Medicine, Addenbrooke’s Hospital, University of Cambridge, Cambridge, United Kingdom; b Division of Medicinal Chemistry, University of Amsterdam, Amsterdam, The Netherlands; University of Michigan—Ann Arbor

**Keywords:** cytomegalovirus, immunotherapy, latent infection, virology

## Abstract

Reactivation of human cytomegalovirus (HCMV) latent infection from early myeloid lineage cells constitutes a threat to immunocompromised or immune-suppressed individuals. Consequently, understanding the control of latency and reactivation to allow targeting and killing of latently infected cells could have far-reaching clinical benefits. *US28* is one of the few viral genes that is expressed during latency and encodes a cell surface G protein-coupled receptor (GPCR), which, during lytic infection, is a constitutive cell-signaling activator. Here we now show that in monocytes, which are recognized sites of HCMV latency *in vivo*, US28 attenuates multiple cell signaling pathways, including mitogen-activated protein (MAP) kinase and NF-κB, and that this is required to establish a latent infection; viruses deleted for US28 initiate a lytic infection in infected monocytes. We also show that these monocytes then become potent targets for the HCMV-specific host immune response and that latently infected cells treated with an inverse agonist of US28 also reactivate lytic infection and similarly become immune targets. Consequently, we suggest that the use of inhibitors of US28 could be a novel immunotherapeutic strategy to reactivate the latent viral reservoir, allowing it to be targeted by preexisting HCMV-specific T cells.

## IMPORTANCE

Human cytomegalovirus (HCMV) is a betaherpesvirus and a leading cause of morbidity and mortality among immunosuppressed individuals. HCMV can establish latent infection, where the viral genome is maintained in an infected cell, without production of infectious virus. A number of genes, including *US28*, are expressed by HCMV during latent infection. US28 has been shown to activate many cellular signaling pathways during lytic infection, promoting lytic gene expression and virus production. As such, the role of US28 remains unclear and seems at odds with latency. Here, we show that US28 has the opposite phenotype in cells that support latent infection—it attenuates cellular signaling, thereby maintaining latency. Inhibition of US28 with a small-molecule inhibitor causes HCMV latent infection to reactivate, allowing latently infected cells to be detected and killed by the immune system. This approach could be used to treat latent HCMV to clear it from human transplants.

## INTRODUCTION

Human cytomegalovirus (HCMV) is a betaherpesvirus which has characteristic lytic and latent stages as part of its lifecycle (1). It is a widespread pathogen, establishing lifelong infection in 50% to 90% of the population (2). Due to robust host T cell and antibody immune responses, primary infection with HCMV is rarely symptomatic in healthy individuals. Despite this, HCMV infection is never cleared after primary infection but persists for the lifetime of the host; this is due, at least in part, to the ability of the virus to establish a latent infection which helps support immune evasion (3). Although primary infection, as well as sporadic reactivation from latency, is asymptomatic in healthy individuals, it can be a severe clinical threat in immunocompromised individuals, such as transplant recipients and patients with AIDS (4).

Although many cell types become lytically infected upon primary HCMV infection (5), only cells of the early myeloid lineage have been shown to carry latent virus *in vivo*. These include CD34^+^ progenitor cells as well as their derivative CD14^+^ monocytes (4). In these cells, the viral genome is maintained with a limited latency-associated transcription program which does not support the production of infectious virus. Differentiation of these latently infected early myeloid lineage cells to macrophages or dendritic cells (DCs) triggers the lytic transcription program and full virus reactivation (6–12). It is now relatively well established that the differentiation-dependent reactivation of HCMV in the myeloid lineage is associated with changes in posttranslational modifications of histones around the major HCMV lytic promoter, namely, the major immediate early promoter (MIEP). These changes drive expression of the major lytic IE72 and IE86 viral gene products, thereby initiating the lytic transcription program and the production of infectious virions (7, 9, 12–17). While the exact signals associated with myeloid differentiation which induce HCMV reactivation are far from clear, it is becoming apparent that orchestrated effects of both cellular and viral factors are involved in this derepression of the MIEP and induction of the lytic transcription program (15, 18–22) and that these effects are likely to involve extracellular signal-regulated kinase–mitogen-activated protein kinase (ERK-MAP kinase) signaling (23). Consequently, these pathways, which are activated during myeloid cell differentiation and maturation, are likely to play a prominent role in the differentiation-dependent activation of the MIEP (18–20, 24–28).

Besides repression of the MIEP and expression of specific latency-associated viral gene products (29–36), latent HCMV carriage has been shown to cause changes in the cellular microRNAome (37, 38), the cellular secretome (39), and cell surface protein expression (40); all of these are likely to be mediated by expression of latency-associated genes (41). One of these, US28, is a G protein-coupled receptor (GPCR) and chemokine receptor (CCR) homologue whose expression has been detected in both natural and experimental models of HCMV latency (30, 31, 35, 42). US28 is one of four HCMV-encoded CCR homologues and is the only one to be expressed during both lytic and latent infection; the other CCR homologues, UL33, UL78, and US27, are expressed only during lytic infection (43–45). Supporting this assertion, deletion of the US27, UL33, and UL78 genes does not affect the establishment of HCMV latency, whereas deletion of the US28 gene has profound effects on latent infection in CD34^+^ progenitor cells and leads to lytic infection in these undifferentiated myeloid cells, due to a lack of MIEP repression (21).

US28 is the best characterized of the CCR homologues encoded by HCMV, and its structure has recently been solved (46). This viral GPCR can signal via multiple different G-alpha proteins, and this signaling is modulated by cell type and cytokine binding (47–51) to activate a number of different signaling pathways (52–59). US28 signaling can be modulated by binding of either CC or CX3C chemokines (52, 59–61), and high-affinity chemokine binding to US28 is known to be mediated by its N-terminal domain (51); one point mutation to US28, US28-Y16F, greatly reduces chemokine binding for RANTES and fractalkine (59).

During lytic infection, US28 is known to promote proliferative signals, including MAP kinase and NF-κB (48, 49, 57, 62), both of which are known to activate the MIEP (63, 64), and its expression has also been linked to vascular disease and oncomodulation (52, 65, 66). This signaling by US28 during lytic infection requires G protein binding via the highly conserved DRY motif of US28 that is found in most GPCRs and all CCRs (67); consistent with this, a point mutation in this DRY motif of US28 (US28-R129A) greatly reduces G-protein binding and ablates US28 signaling capability (48, 54, 68, 69). Similarly, the US28 C terminus is also heavily phosphorylated and this is also known to modulate US28 signaling (70–72). All these known activator functions of US28 during lytic infection, however, appear to be totally inconsistent with the observation that US28 is required to enforce MIEP silencing during latency (21) and suggest very different functions of US28 during latent infection and lytic infection.

In this work, we address this issue in detail by analyzing the effects of US28 seen during HCMV latency. First, we show that, as with CD34^+^ cells, US28 expression is also necessary for the maintenance of HCMV latency in CD14^+^ monocytes and that this US28 activity is not dependent on US28 binding of chemokines but requires constitutive G protein-coupled signaling. We also show that US28 has a signaling profile in undifferentiated monocytic cells that is completely different from that in differentiated, macrophage-like cells; in undifferentiated myeloid cells, US28 attenuates multiple different cell signaling pathways, including MAP kinase and NF-κB signaling, and it is the US28-mediated repression of these signaling pathways which helps US28 to repress the MIEP, thereby maintaining latency. Consistent with this, treatment of latently infected cells with a small-molecule inhibitor of US28 resulted in induction of IE expression and virus release. Finally, we demonstrate that monocytes infected with HCMV lacking US28 or treated with US28 inverse agonists and which consequently express viral IE proteins are recognized and killed by preexisting HCMV-specific T cells from HCMV-seropositive donors and propose that small-molecule inhibition of US28 could be a novel shock-and-kill approach for targeting latent HCMV for existing host T cell responses.

## RESULTS

### US28 is required for HCMV to establish latency in monocytes.

**FIG 1:**
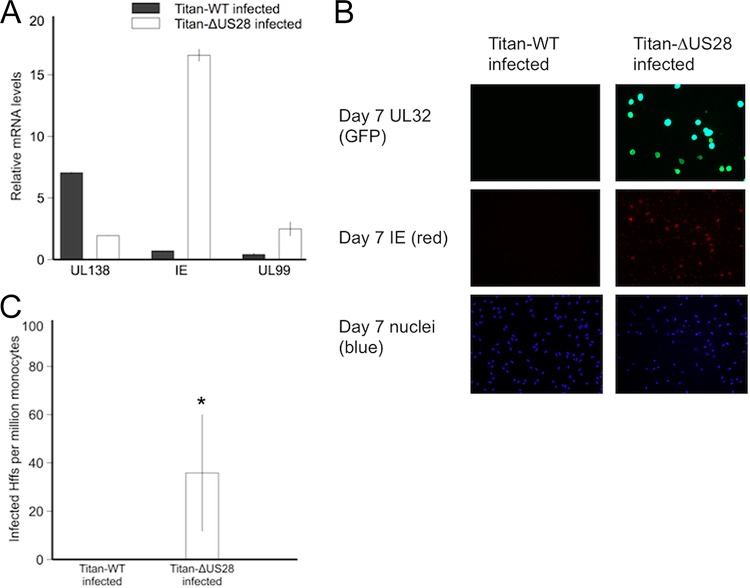
Infection of monocytes with Titan-ΔUS28 results in lytic infection. CD14^+^ peripheral blood monocytes were isolated and experimentally infected at an MOI of 5 with HCMV Titan-WT or Titan-ΔUS28. (A) Seven days postinfection, RNA from these cultures was harvested and analyzed for expression of the latency-associated UL138 gene, the major immediate early lytic IE1 gene, and the major late UL99 gene. Data were normalized to GAPDH RNA. (B) Seven days postinfection, monocytes were also fixed and stained for IE protein and UL32-GFP (using an antibody against the GFP tag). (C) These monocyte cultures were then cocultured with HFFs, and the number of infected HFFs was measured by staining for cells expressing HCMV IE protein at 72 h postcoculture. All data points show means of results from at least four independent experiments; error bars show standard deviations. *, *P* = 0.05 (statistically significant result; calculated using Student’s *t* test).

Latent infection with HCMV is characterized by the expression of latency-associated genes, by little concomitant lytic immediate early (IE) gene expression (32), and by the absence of production of infectious virions. As expected, infection of monocytes after 7 days with a clinical isolate of HCMV, the wild-type Titan strain (Titan-WT), which has a UL32-green fluorescent protein (UL32-GFP) tag that is expressed only at late times of lytic infection, resulted in a characteristically latent infection—with high levels of expression of viral UL138, a known latency-associated transcript, and with little accompanying IE72 or late UL99 RNA (Fig. 1A). Similarly, these Titan-WT-infected cells did not show any IE72 or late UL32-GFP protein expression (Fig. 1B, left panels) and did not produce infectious virions in analyses performed by coculture on indicator fibroblasts (Fig. 1C). In contrast, infection of monocytes with a virus in which US28 had been deleted (Titan-ΔUS28) showed high levels of IE72 RNA and concomitant expression of UL99 RNA, a true late gene transcript (Fig. 1A), when infected with similar amounts of virus, as assessed by genome copy quantitative PCR (qPCR) analysis (see Fig. S1 in the supplemental material). To confirm the expression of IE72 and late proteins in these cells, we also stained the infected monocytes for IE72 protein and the late gene UL32-GFP protein and observed clear evidence of expression of IE and UL32 proteins (Fig. 1B, right panels). Finally, we cocultured these monocytes with indicator fibroblasts to quantify any virus release and also observed the presence of infectious virus in monocytes infected with Titan-ΔUS28 virus but not in those infected with Titan-WT virus (Fig. 1C). Consistent with viral lytic replication occurring only in Titan-ΔUS28-infected monocytes, increases in viral genome copy numbers were detected in Titan-ΔUS28-infected monocytes but not Titan-WT-infected monocytes 7 days postinfection (Fig. S1).

10.1128/mBio.01754-17.1 FIG S1  Titan-ΔUS28-infected monocytes replicate viral genomes. CD14^+^ peripheral blood monocytes were isolated from the PBMCs of healthy donors and experimentally infected at an MOI of 5 with Titan-WT or Titan-ΔUS28. At either 1 day or 7 days postinfection, DNA was collected from cultures and genomes were quantified by qPCR analysis of the MIEP region. Values were corrected to GAPDH qPCR values; all data points show means of results from three independent experiments; error bars show standard deviations. Download FIG S1, TIF file, 2.1 MB. Copyright © 2018 Krishna et al. 2018 Krishna et al. This content is distributed under the terms of the Creative Commons Attribution 4.0 International license.

Taken together, these data argue for a requirement for US28 in either establishment or maintenance of latent infection of CD14^+^ monocytes and show that, in the absence of US28, monocytes undergo full lytic infection.

### Lytic infection of monocytes by Titan-ΔUS28 virus does not result from early induction of myeloid differentiation.

**FIG 2:**
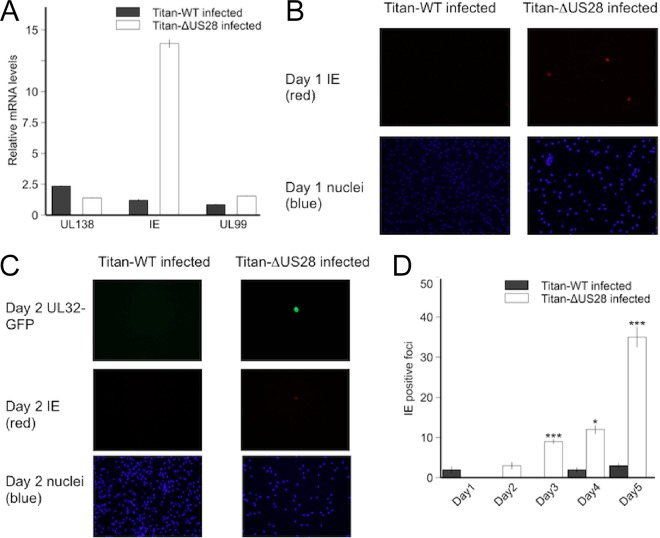
Titan-ΔUS28 virus initiates lytic infection immediately after infection of CD14^+^ monocytes. CD14^+^ peripheral blood monocytes were isolated and experimentally infected at an MOI of 5 with HCMV Titan-WT or Titan-ΔUS28. (A) Twelve hours postinfection, RNA from these cultures was harvested and analyzed by RT-qPCR for the latent UL138 gene and the lytic IE and UL99 genes. Data were normalized to GAPDH RNA. (B) One day postinfection, monocytes were fixed and stained for IE protein. (C) Two days postinfection, monocytes were fixed and stained for IE or UL32-GFP protein. (D) Each day postinfection, medium was harvested from monocytes infected with Titan-WT or Titan-ΔUS28 and titrated onto indicator HFFs. These were subsequently stained for HCMV IE protein, as a measure of viral titers, 24 h postinfection. All data points show means of results from at least four independent experiments; error bars show standard deviations.

Having established that infection of CD14^+^ monocytes with Titan-ΔUS28 for 7 days resulted in a lytic rather than a latent infection, we wanted to rule out the possibility that US28 was simply maintaining a latent infection by actively suppressing myeloid cell differentiation. We reasoned that if this were the case, and if an absence of US28 simply induces infected monocytes to differentiate and to become fully permissive for HCMV lytic infection, there ought to be a temporal delay in the induction of lytic gene expression in monocytes infected with Titan-ΔUS28 to allow time for differentiation to occur. Consequently, we assayed for IE mRNA and protein expression in monocytes infected with Titan-WT or Titan-ΔUS28 but at very early time points postinfection. These analyses clearly showed that substantial levels of lytic IE gene expression were observed as early as 12 h postinfection of monocytes with Titan-ΔUS28 (Fig. 2A). We were also able to detect IE protein expression by immunofluorescent (IF) microscopy from as early as 24 h postinfection (Fig. 2B) and UL32-GFP expression from 48 h postinfection (Fig. 2C). Additionally, supernatants from Titan-ΔUS28-infected monocytes, at 3 days postinfection, showed the presence of infectious virus on indicator fibroblasts (Fig. 2D). To further support those experiments, we performed flow cytometry analysis on monocytes infected with Titan-WT or Titan-ΔUS28 for 7 days and observed little change in cell surface expression of CD14 or CD83, each of which is a marker of differentiation (Fig. S2). All these data were entirely consistent with the view that Titan-ΔUS28-infected monocytes immediately undergo a lytic infection with little or no temporal delay and make it unlikely that infection in the absence of US28 simply induced differentiation of monocytes to a cell phenotype that is permissive for lytic infection.

10.1128/mBio.01754-17.2 FIG S2  CD14^+^ monocytes infected with Titan-ΔUS28 showed no changes in phenotypic markers associated with myeloid differentiation. CD14^+^ peripheral blood monocytes were isolated from the PBMCs of healthy donors and experimentally infected at an MOI of 5 with Titan-WT or Titan-ΔUS28. At 7 days postinfection, cells were stained with anti-CD14 (A) or anti-CD83 (B) antibodies and analyzed by flow cytometry. Download FIG S2, TIF file, 1.8 MB. Copyright © 2018 Krishna et al. 2018 Krishna et al. This content is distributed under the terms of the Creative Commons Attribution 4.0 International license.

### US28 signaling maintains latency independently of chemokine binding.

**FIG 3:**
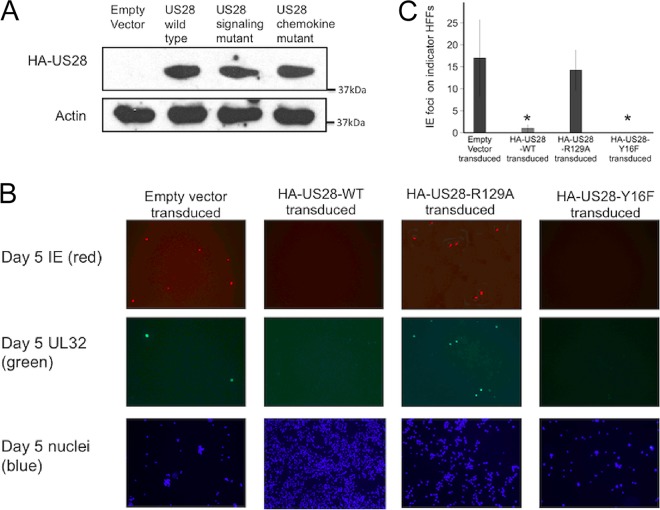
Ectopic US28 expression in THP-1 cells can complement for a deletion of US28 from the virus. THP-1 cells stably expressing N-terminally HA-tagged US28 (HA-US28-WT), US28 with a disrupted G protein binding DRY motif (HA-US28-R129A), and US28 with a disrupted chemokine binding region (HA-US28-Y16F) were generated by lentiviral transduction and puromycin selection. (A) Western blot analysis using an antibody against the N-terminal HA tag was carried out on an empty-vector-transduced cell line and the three cell lines expressing HA-US28 constructs. (B) These THP-1 cells, expressing different HA-US28 constructs and empty vector control cells, were infected with Titan-ΔUS28 and fixed 5 days postinfection. Fixed samples were stained for immediate early or UL32-GFP and nuclei were also stained. (C) Media from these infected cells was titrated on indicator fibroblasts and the number of infectious virions quantified by IE staining. Data are means of results from at least three independent experiments; error bars show standard deviations.

On the basis of the observation that US28 appeared to be having a profound effect on the outcome of infection of monocytes, supporting at least in part the establishment of latency, we next decided to assess the effects of US28 expression on monocytic cells in detail. To do this, we used lentiviral vectors to overexpress an N-terminally hemagglutinin (HA)-tagged US28 (HA-US28-WT) (73) in isolation in the monocytic THP-1 cell line which we and others have used as a model of latent HCMV infection (42, 74, 75). At the same time, we also overexpressed two HA-tagged US28 mutants, namely, HA-US28-R129A and HA-US28-Y16F, which have ablated signaling and chemokine binding function, respectively (Fig. 3A).

We then infected these THP-1 cell lines, stably expressing HA-US28-WT, HA-US28-R129A, or HA-US28-Y16F proteins, with Titan-ΔUS28 virus to assess whether supplying these US28 proteins in *trans* would affect the ability of Titan-ΔUS28 to undergo a lytic infection in these undifferentiated monocytic cells.

Figure 3B shows that, as expected, control THP-1 cells stably transduced with an empty vector underwent lytic infection when infected with Titan-ΔUS28 virus, in that IE and UL32-GFP proteins were detectable. In contrast, expression of HA-US28-WT in THP-1 cells complemented the lack of US28 in Titan-ΔUS28 virus and this resulted in cells negative for IE and UL32-GFP expression—consistent with a latent infection. Interestingly, THP-1 cells expressing the HA-US28-R129A protein failed to complement the Titan-ΔUS28 virus mutation (these infected cells were IE and UL32-GFP positive), whereas infection of THP-1 cells stably expressing the HA-US28-Y16F mutant also complemented Titan-ΔUS28 virus and resulted in cells undergoing latent infection (as detected by a lack of IE and UL32-GFP expression) (Fig. 3B). Also as expected, THP-1 cells infected with Titan-WT showed little lytic gene expression, regardless of expression of any HA-US28 construct (Fig. S3).

10.1128/mBio.01754-17.3 FIG S3  Ectopic US28 expression in THP-1 cells does not affect the establishment of latency under conditions of infection with Titan-WT virus. THP-1 cells stably expressing HA-US28-WT, HA-US28-R129A, or HA-US28-Y16F (see Fig. 3) were infected with Titan-WT for 5 days. Cells were then fixed and stained for IE proteins or UL32-GFP, and nuclei were also stained. Download FIG S3, TIF file, 1.5 MB. Copyright © 2018 Krishna et al. 2018 Krishna et al. This content is distributed under the terms of the Creative Commons Attribution 4.0 International license.

We also tested whether any observed failure to complement Titan-ΔUS28 by these US28 constructs, which resulted in lytic gene expression, also resulted in production of infectious virus. Figure 3C shows that cells in which IE and late gene expression could be detected also produced infectious virions, as expected.

Finally, we confirmed that the ability of HA-US28-WT and HA-US28-Y16F to complement Titan-ΔUS28 and to establish latent infection resulted in cells from which HCMV could be reactivated by differentiation (Fig. S4). Taken together, these data suggest that the ability of US28 to suppress lytic infection likely resides in its downstream signaling, via G protein activation, and that this signaling occurs independently from chemokine binding.

10.1128/mBio.01754-17.4 FIG S4  Ectopic US28 expression in THP-1 cells complements for a deletion of US28, and virus can be reactivated from these cells. THP-1 cells stably expressing HA-US28-WT, HA-US28-R129A, or HA-US28-Y16F (see Fig. 3) were infected for 3 days with Titan-ΔUS28 and then subsequently treated with PMA. At 4 days post-PMA treatment, cells were fixed and stained for immediate early or UL32-GFP and nuclei were also stained. Download FIG S4, TIF file, 1.9 MB. Copyright © 2018 Krishna et al. 2018 Krishna et al. This content is distributed under the terms of the Creative Commons Attribution 4.0 International license.

### US28 suppresses or activates the MIEP depending on the differentiation status of the monocytic cell.

**FIG 4:**
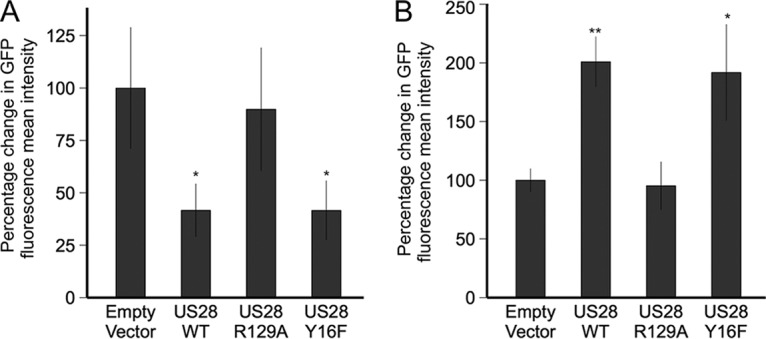
US28 represses the MIEP in undifferentiated myeloid cell lines but activates it in differentiated myeloid cells. (A) THP-1 cells which had been transduced with an MIEP-eGFP construct were then transfected by nucleofection with HA-US28-WT, HA-US28-R129A, or HA-US28-Y16F constructs. Three days after nucleofection, cells were analyzed for eGFP expression by flow cytometry. (B) Additionally, cells were treated with PMA 2 days after nucleofection and were analyzed by flow cytometry 2 days after treatment. Data show percentages of change in mean fluorescent intensities from four technical replicates, after selection for single cells and exclusion of dead cells using Zombie red dye. Error bars show standard deviations. *P* values of 0.05 (*) and 0.01 (**) were calculated using Student’s *t* test and were considered significant.

As US28 signaling appeared to be necessary for the establishment of latency in monocytes, we hypothesized that US28 expression likely negatively regulates the MIEP in undifferentiated monocytic cells. To test this, we used THP-1 cell lines that had been transduced with an MIEP-enhanced GFP (MIEP-eGFP) construct (76) to transfect these cells by nucleofection with three HA-US28 constructs and with the empty vector control (Fig. S5). Two days posttransfection, we measured eGFP expression in these cell lines by flow cytometry. Figure 4A shows that, consistent with a role for suppression of lytic infection in undifferentiated THP-1 cells, HA-US28-WT did, indeed, show repression of MIEP activity, as did the HA-US28-Y16F mutant. In contrast, the HA-R129A-US28 signaling mutant showed no such repression. We also repeated this analysis, but, 2 days after nucleofection with the HA-US28 constructs and empty vector control, we differentiated the THP-1 cells with phorbol myristate acetate (PMA) (Fig. 4B). In contrast to the results seen with undifferentiated THP-1 cells, HA-US28-WT and HA-US28-Y16F now activated the MIEP, whereas HA-US28-R129A expression had no significant effect on MIEP activity. These data confirm that the effect of US28 on IE gene expression is differentiation dependent; US28 appears to repress the MIEP in undifferentiated monocytic cells, consistent with a role of US28 in maintaining latency, but activates the MIEP after cellular differentiation, likely to promote lytic infection.

10.1128/mBio.01754-17.5 FIG S5  US28 represses the MIEP in undifferentiated myeloid cell lines but activates it in differentiated myeloid cells. THP-1 cells which had been transduced by lentivirus to stably express an MIEP-eGFP construct were then transfected by nucleofection with HA-US28-WT, HA-US28-R129A, or HA-US28-Y16F constructs. Western blot analysis using an antibody against the N-terminal HA tag was carried out on an empty-vector-transfected cell line, and the three cell lines were transfected with HA-US28 constructs. Download FIG S5, TIF file, 0.2 MB. Copyright © 2018 Krishna et al. 2018 Krishna et al. This content is distributed under the terms of the Creative Commons Attribution 4.0 International license.

### US28 attenuates MAP kinase and NF-κB cell signaling pathways.

**FIG 5:**
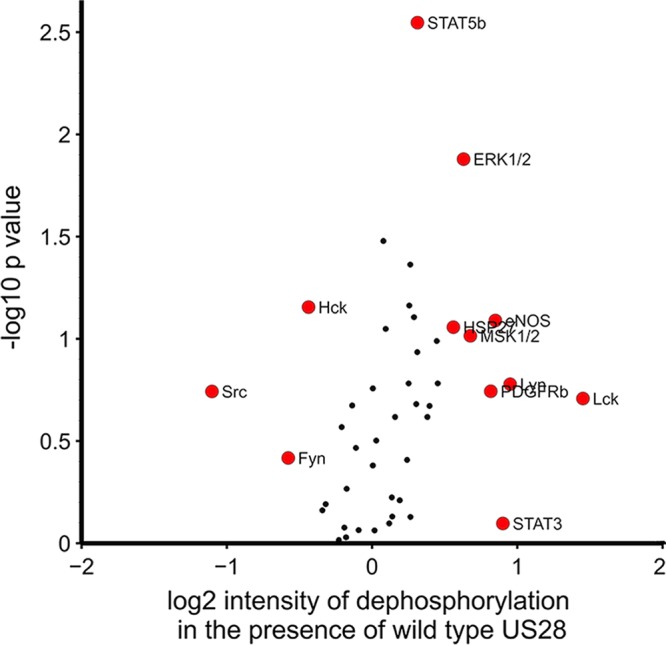
US28 expression in THP-1 cells, in isolation, attenuates cellular signaling. THP-1 cells which had been induced to express either US28-WT or US28-R129A (which cannot maintain latency) were lysed and analyzed for changes in cellular kinase phosphorylation levels by antibody array. Data represent fold change in dephosphorylation of each kinase from THP-1 cells expressing US28-WT over the levels induced by THP-1 cells expressing US28-R129A. Data points in red represent results that showed a change in intensity of ± log_2_ (0.5) arbitrary units and/or a *P* value greater than log_10_ (1.5).

US28 expression during lytic infection is known to activate a number of cell signaling pathways, including the NF-κB and MAP kinase pathways, both of which are known to activate the MIEP in fully permissive cells. To analyze the potential effect of US28 on such signaling during latent infection, we used phosphokinase antibody arrays to assess whether US28 mediates changes in phosphorylation of an array of different cellular signaling proteins (Fig. 5). Specifically, we compared THP-1 cells expressing either HA-US28-WT or the HA-US28-R129A signaling mutant; we reasoned that comparing HA-US28-WT to the HA-US28-R129A signaling mutant would robustly control for potential nonspecific effects of US28 protein overexpression. These analyses showed that HA-US28-WT specifically decreased the phosphorylation of a number of cellular proteins, suggesting a general attenuation of cell signaling pathways by wild-type US28 in undifferentiated monocytic cells. In particular, we noted significant reductions in the phosphorylation levels of several key signaling proteins, chief among them being ERK1/2 of the MAP kinase pathway (Fig. 5).

**FIG 6:**
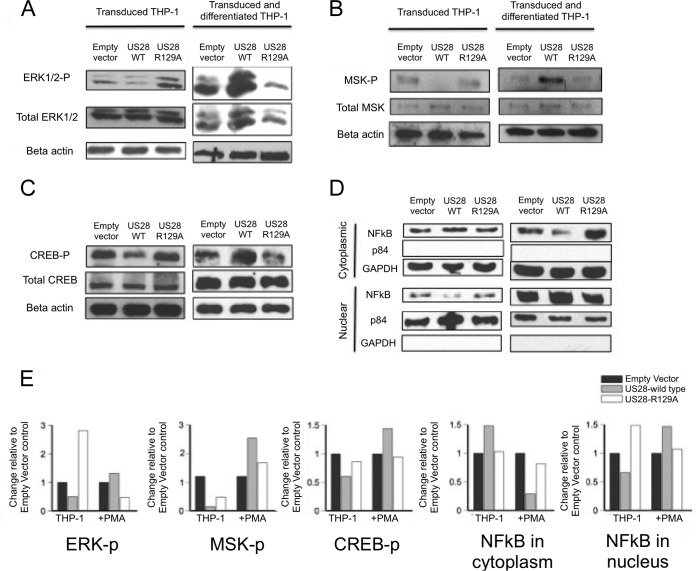
US28 expression in THP-1 cells, in isolation, attenuates MAP kinase and NF-κB cellular signaling. (A) THP-1 cells expressing either HA-US28-WT or HA-US28-R129A (which cannot maintain latency) or an empty vector control were lysed and analyzed by Western blotting for phospho- and total ERK1/2 and beta-actin levels (left panels). THP-1 cells were also differentiated with PMA treatment and, at 4 days posttreatment, were analyzed by Western blotting for phospho- and total ERK1/2 and beta-actin levels (right panels). (B and C) The same analysis was performed for phospho- and total MSK-1 in undifferentiated cells (B, left panels) or 4 days after differentiation with PMA treatment (B, right panels) and also for phospho- and total CREB (C). (D) Cells were also fractionated into nuclear and cytoplasmic fractions, and these fractions were subjected to Western blot analysis for NF-κB protein (p65) before (left) and after (right) differentiation. Nuclear protein p84 and cytoplasmic protein GAPDH were used as loading and fractionation controls for the respective fractions. (E) These blots were subjected to densitometry analysis using ImageJ software, and the relative amounts of phosphoprotein (for panels A to C) or NF-κB localization (for panel D) were quantified against actin, GAPDH, or p84 loading controls.

Our result, showing that US28 mediated suppression of MIEP activity as well as inhibiting ERK1/2, fits well with the view that MAP kinase signaling is likely involved in HCMV reactivation (23). Consequently, we validated the results of the phosphokinase array by performing Western blotting on three cellular proteins that are key to the MAP kinase signaling pathway: ERK1/2, mitogen and stress activated kinase (MSK-1), and cAMP response binding element-binding protein (CREB). Figure 6A to C (left panels) shows that all three proteins were hypophosphorylated in THP-1 cells expressing HA-US28-WT compared to control cells expressing HA-US28-R129A. As US28 is associated with activation of MAP kinase during lytic infection, we also repeated this analysis in these THP-1 cells after they had been differentiated to a macrophage-like phenotype (which is permissive with respect to HCMV lytic infection) (6,8,9,12). Figure 6 shows that overexpression of US28 now had the opposite effect on ERK1/2, MSK-1, and CREB phosphorylation; HA-US28-WT protein resulted in their hyperphosphorylation compared to the results seen with cells expressing HA-US28-R129A (Fig. 6A to C).

Finally, we performed nuclear/cytoplasmic fractionation followed by Western blotting to analyze the effect of US28 on NF-κB activation (Fig. 6D). These analyses showed that the NF-κB pathway is also attenuated by US28 in undifferentiated monocytic THP-1 cells in that, in contrast to the results seen with HA-US28-R129A or control vector, US28 expression resulted in a relative lack of nuclear localization of p65 (Fig. 6D, left panels). As predicted, this was reversed in differentiated THP-1 cells (Fig. 6D, right panels).

### Inhibition of MAP kinase and NF-κB cell signaling pathways can reduce lytic infection of monocytes by Titan-ΔUS28 virus.

**FIG 7:**
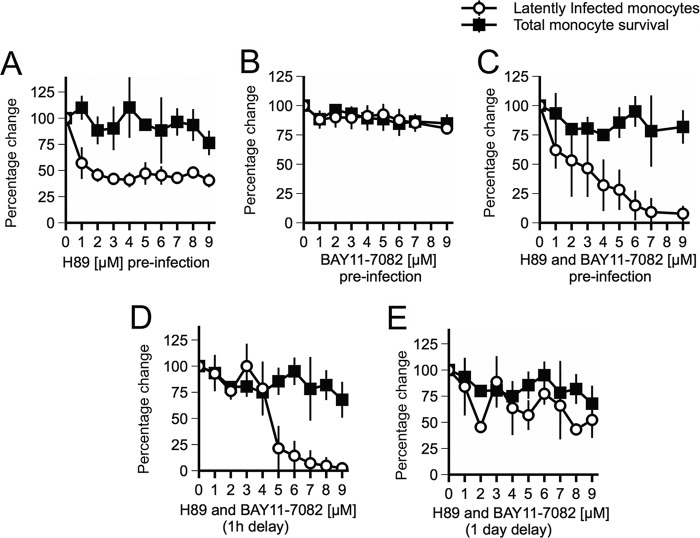
Inhibition of MAP kinase and NF-κB pathways can prevent lytic infection of monocytes by Titan-ΔUS28. (A) CD14^+^ peripheral blood monocytes were isolated and infected at an MOI of 5 with Titan-ΔUS28 in the presence of increasing concentrations of H89 (an inhibitor of MSK-1). Subsequently, GFP-positive cells were counted 3 days postinfection and cell survival was measured by trypan blue exclusion staining. (B) Monocytes were infected with Titan-ΔUS28 in the presence of titrations of BAY11-7082 (an inhibitor of IKKα). Subsequently, GFP-positive cells were counted 3 days postinfection and cell survival was measured by trypan blue exclusion staining. (C) Monocytes were treated with both H89 and BAY11-7082 and then infected with Titan-ΔUS28 in the presence of both inhibitors. Subsequently, GFP-positive cells were counted 3 days postinfection and cell survival was measured by trypan blue exclusion staining. (D) Monocytes were infected with Titan-ΔUS28, but treatment with H89 and BAY11-7082 was delayed until 1 h postinfection. Subsequently, GFP-positive cells were counted 3 days postinfection and cell survival was measured by trypan blue exclusion staining. (E) Monocytes were infected with Titan-ΔUS28, but treatment with H89 and BAY11-7082 was delayed until 1 day postinfection. Subsequently, GFP-positive cells were counted 3 days postinfection and cell survival was measured by trypan blue exclusion staining. All data points show means of results from at least three independent experiments; error bars show standard deviations.

On the basis of the findings that US28 expression, in isolation in undifferentiated myeloid cells, attenuates MAP kinase and NF-κB signaling pathways and that this correlates with the suppression of the MIEP and the ability of HCMV to establish latency, we reasoned that we should be able to mimic the action of US28 in undifferentiated CD14^+^ monocytes by inhibiting MAP kinase and/or NF-κB signaling, in the context of HCMV infection; in essence, we reasoned that we could compel Titan-ΔUS28 virus to establish latency in monocytes by pretreating these monocytes with inhibitors of either MSK-1 or IκB kinase (IKKα) (using H89 or Bay11-7082, respectively) before infection. To test this, we treated cells with inhibitors prior to infection with Titan-ΔUS28 and then measured the number of UL32-GFP-positive cells 3 days postinfection, as an indicator of full, lytic infection. Figure 7A and B show that neither inhibitor alone was able to prevent Titan-ΔUS28 virus from undergoing lytic infection. However, infection in the presence of both inhibitors together did, indeed, lead to an absence of UL32-GFP gene expression in Titan-ΔUS28-infected monocytes in a dose-dependent manner and this could not be attributed to nonspecific cell toxicity effects (Fig. 7C, black squares).

Our observations presented in Fig. 2 indicating that Titan-ΔUS28 virus initiated IE expression in infected monocytes 6 to 12 h postinfection also suggested that US28 may be required at very early times of HCMV infection of monocytes to suppress the MIEP and help establish latency. Therefore, we hypothesized that, during infection with HCMV virus, US28 protein expression likely blocks activation signals for the MIEP in monocytes, which could occur due to virus binding or internalization. In order to test this, we delayed inhibitor treatment of monocytes infected with Titan-ΔUS28 until either 1 h or 1 day postinfection, assuming that delaying inhibition of MAP kinase and NF-κB would lead to the activation of the MIEP by viral entry and that this would no longer be preventable once MIEP activity was fully established. Treatment of monocytes with H89 and BAY11-7082 at 1 h postinfection was still effective at blocking lytic infection by Titan-ΔUS28. However, when treatment was delayed until 1 day postinfection, the inhibitors were no longer as effective at blocking lytic infection (Fig. 7D and E). These data suggest that signals triggered within the first 24 h of infection of monocytes by HCMV activate MIEP activity but that US28 attenuates these signals to suppress IE expression in order to establish latent infection.

### US28 attenuation of cellular signaling prevents phosphorylation of histone H3 and subsequent activation of the MIEP.

**FIG 8:**
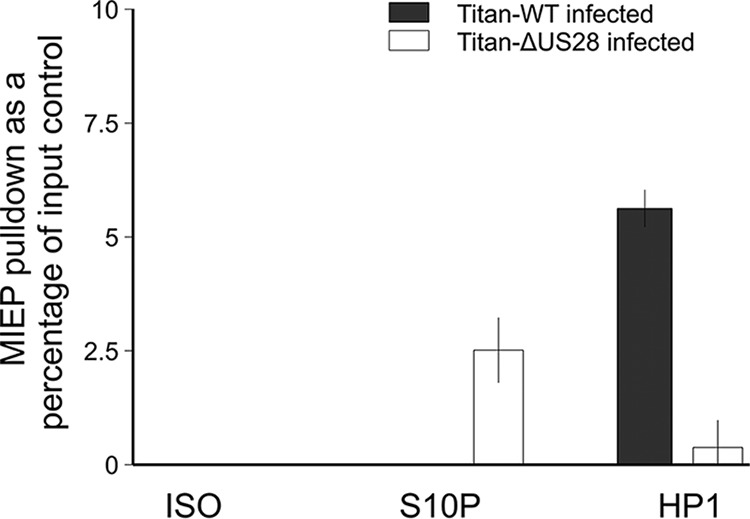
The MIEP is associated with phosphorylated serine-10-H3 in the absence of US28. CD14^+^ monocytes were infected with either Titan-WT or Titan-ΔUS28. The cells were harvested 6 days postinfection for ChIP analysis (using a Sigma Imprint ChIP kit). The MIEP was precipitated with either anti-HP1 (HP1 antibody [FL-191]: sc-28735) or anti-ser-10-H3 (phospho-histone H3 [Ser10] antibody [9H12L10], ABfinity Rabbit Monoclonal) or the isotype control (ISO). The MIEP was quantitated against a standard curve of viral DNA and analyzed by qPCR, and the results were then plotted as a percentage of input DNA, with each sample run in triplicate.

It is well established that in undifferentiated myeloid cells, the viral MIEP is associated with histone marks of transcriptional repression, including the presence of methylated histones and repressor proteins such as heterochromatin protein 1 (HP1). In contrast, when this repression is relieved during myeloid differentiation, the MIEP becomes associated with histone marks of transcriptional activation such as the presence of acetylated histones and histone H3 phosphorylation (7, 9, 12–17). It has also been established, more recently, that myeloid differentiation activates CREB binding to the MIEP, causing corecruitment of MSK-1 and subsequent phosphorylation of histone H3, which is known to destabilize the binding of HP1 (23). We therefore hypothesized that US28-mediated attenuation of MSK-1 and CREB signaling could prevent histone phosphorylation and activation of the MIEP, thereby maintaining latency. Consequently, we infected monocytes with Titan-WT and Titan-ΔUS28 and performed chromatin immunoprecipitation (ChIP) assays against HP-1 and H3-S10p, markers of histone repression and activation, respectively. As expected, the MIEP was associated with HP-1 in monocytes latently infected with Titan-WT but was associated with phosphorylated H3 in monocytes lytically infected with Titan-ΔUS28 (Fig. 8). US28, therefore, appears to mediate parts of its repressive function via prevention of destabilization of HP1 binding.

### The US28 inhibitor VUF2274 can induce lytic infection in monocytes infected with wild-type HCMV.

**FIG 9:**
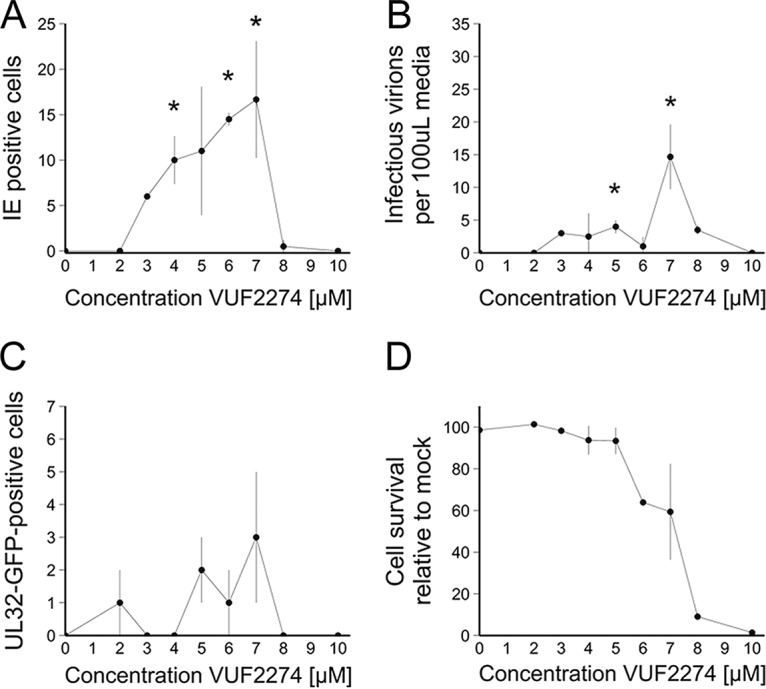
VUF2274 is able to induce reactivation of HCMV from latently infected monocytes. CD14^+^ peripheral blood monocytes were isolated and infected at an MOI of 5 with RV1164, which has an IE2-YFP fluorescent tag. Three days postinfection, increasing concentrations of the US28 inhibitor VUF2274 were added to cells. (A) IE2-YFP-positive cells were counted by immunofluorescent microscopy at 72 h posttreatment. (B) Five days post-drug treatment, medium was removed from these cells and titrated onto HFFs. Three days postinfection, HFFs were fixed and stained for IE protein, and IE-positive cells were counted by immunofluorescence microscopy. (C) CD14^+^ peripheral blood monocytes were isolated and infected at an MOI of 5 with Titan-WT. Three days postinfection, increasing concentrations of the US28 inhibitor VUF2274 were added to cells. Five days post-drug treatment, UL32-GFP-positive cells were counted by fluorescence microscopy. (D) Cell survival of monocytes in the presence of VUF2274 for 4 days was measured using trypan blue exclusion staining. All data points show means of results from three replicates, and error bars show standard deviations. Data were subjected to analysis of variance (ANOVA) followed by Tukey’s *post hoc* test. *, *P* = 0.05 (statistically significant result).

VUF2274 (BX 513 hydrochloride) is an antagonist of CCR1 and an inverse agonist of US28 (77). Given that our results obtained so far showed that US28 signaling was required to help establish latency in monocytes, we predicted that monocytes infected with wild-type HCMV in the presence of VUF2274 would trigger viral lytic gene expression and, possibly, virus reactivation. To test this, we latently infected monocytes for 3 days with RV1164, an isolate of HCMV that has an IE2-yellow fluorescent protein (IE2-YFP) tag, and then treated them with a titration of VUF2274 and quantified IE protein expression by counting YFP-positive monocytes 3 days after drug treatment (Fig. 9A). We also quantified any production of infectious virus in these cultures treated with VUF2274 by removing media from monocytes 3 days post-drug treatment and titrating this media onto indicator fibroblasts (Fig. 9B). As predicted, VUF2274 treatment did induce IE gene expression (Fig. 9A) and resulted in measurable release of virus from these reactivated cells (Fig. 9B). Consistent with this, equivalent experiments using infection with Titan-WT, which has a UL32-GFP tag, also confirmed late gene expression (UL32) in these VUF2274-treated cells (Fig. 9C). Taken together, these data argue that inhibition of US28 signaling by VUF2274 appears to reactivate full lytic gene expression and lytic infection in monocytes latently infected with wild-type HCMV. It should be pointed out, however, that VUF2274 did show significant toxicity (as measured by trypan blue staining) for primary blood monocytes, even at concentrations below an approximate *K*_*i*_ value of 10 μM (Fig. 9D). Additionally, as expected, although attenuation of cellular signaling by US28 can be inhibited by VUF2274, leading to activation of MIEP and IE protein expression, this signaling to MIEP can be blocked by simultaneous inhibition of MSK-1 and NF-kB (Fig. S6).

10.1128/mBio.01754-17.6 FIG S6  Bay11-7082 and H89, the inhibitors of MAP kinase and NF-kB, can block VUF2274-induced IE gene expression in latent cells. CD14^+^ monocytes were infected with IE2-YFP and then treated with inhibitors (VUF2274 in a concentration gradient and Bay11-7082 and H89 at the fixed concentration of 5 µM) as indicated at 24 h postinfection. Three days later, IE-positive cells were enumerated in triplicate wells of a 96-well plate. All data points show means of results from three replicates, and error bars show standard deviations. Data were subjected to analysis of variance (ANOVA) followed by Tukey’s *post hoc* test. *, *P* = 0.05 (statistically significant result). Download FIG S6, TIF file, 23.4 MB. Copyright © 2018 Krishna et al. 2018 Krishna et al. This content is distributed under the terms of the Creative Commons Attribution 4.0 International license.

### Monocytes infected with Titan-ΔUS28 virus are targets for killing by preexisting HCMV-specific donor cytotoxic T cells.

**FIG 10:**
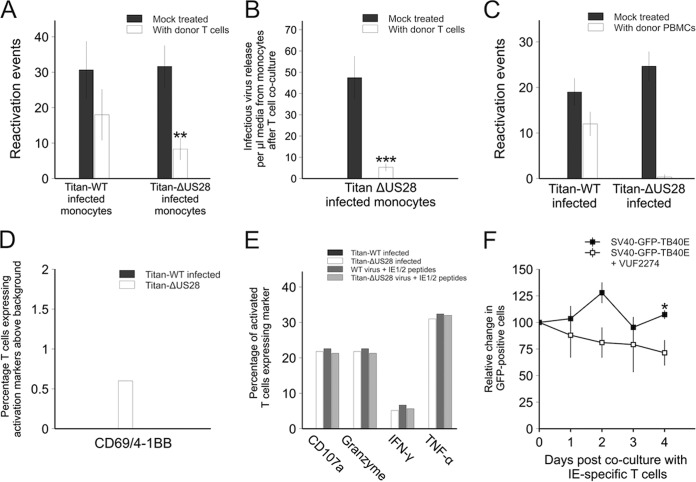
Monocytes infected with Titan-ΔUS28 or monocytes infected with SV40-GFP-TB40E in the presence of US28 inhibitors are targets for HCMV-specific T cell responses. (A) CD14^+^ monocytes were infected with either Titan-WT or Titan-ΔUS28. Three days postinfection, monocytes were cocultured with IE72-specific T cells for a further 3 days. After this, monocytes were washed to remove T cells and monocytes were then differentiated and matured to induce virus reactivation. Reactivated virus was quantified by fibroblast coculture and staining for IE foci. (B) Monocytes were removed from media from the experiment described for panel A at 3 days after T cell treatment and titrated onto fibroblasts to quantify virus release from Titan-ΔUS28-infected monocytes. (C) CD14^+^ monocytes from seropositive donors were infected with either Titan-WT or Titan-ΔUS28, and the nonmonocyte PBMCs were added back to the infected monocytes 3 days postinfection for 4 days and then removed by washing. The remaining adherent monocytes were then differentiated and matured to induce virus reactivation. Reactivated virus was quantified by fibroblast coculture and staining for IE foci. (D) CD14^+^ monocytes from seropositive donors were infected with either Titan-WT or Titan-ΔUS28, and, 5 days postinfection, latent monocytes were left untreated or cocultured overnight with isolated CD8^+^ T cells from the same donor and analyzed for expression of the activation markers CD69 and 4-1BB and for expression of the following degranulation markers: CD107a; granzymes A, B, and K; TNF-α; and IFN-γ. Data in panel D represent the percentage of CD8^+^ T cells expressing both activation markers, above background stimulation levels, in response to either Titan-WT-infected or Titan-ΔUS28-infected monocytes. (E) The proportions of HCMV-specific CD8^+^ T cells expressing degranulation markers seen with those activated monocytes are shown, as are those of CD8^+^ T cells stimulated with virus-infected monocytes and pulsed with IE1/2 peptides as antigen-specific positive controls. (F) Monocytes from an HLA-A2-positive donor were latently infected with SV40-GFP-TB40E and were left untreated or treated with VUF2274 3 days postinfection. Two days posttreatment with drug, monocytes were cocultured with IE72-specific T cells, following which monocytes expressing GFP were counted over the subsequent 4 days. Data points for all panels show means of results from at least three independent experiments; error bars show standard deviations.

It is well established that, after primary infection, healthy HCMV carriers maintain extremely high frequencies of HCMV-specific CD8^+^ cytotoxic T lymphocytes (CTLs) in their peripheral blood which are often dominated by CTLs which recognize IE antigens; up to 10% of effector memory CD8^+^ CTLs can recognize IE72 in some donors (78–80). However, because latently infected cells express only small amounts of these lytic antigens, they escape these HCMV-specific CTL responses. We therefore reasoned that the inability of Titan-ΔUS28-infected monocytes to undergo latent infection, and their resulting high level of lytic gene expression, should make them targets for preexisting HCMV-specific CTLs in the peripheral blood of healthy HCMV carriers. The same would be observed in monocytes latently infected with wild-type virus if US28 were inhibited by VUF2274. Figure 10A shows that coculture of monocytes infected with Titan-ΔUS28 with donor-matched IE72-specific T cell clones resulted in a reduction in the frequency of reactivation of latently infected cells from these infected monocytes after their differentiation and maturation to mature dendritic cells (mDCs). As expected, we also found that treatment with IE-specific T cells resulted in a reduction in the level of virus release from monocytes infected with Titan-ΔUS28, due to killing of these lytically infected cells in the absence of their differentiation and maturation (Fig. 10B). We also repeated this analysis with donor-matched total peripheral blood mononuclear cells (PBMCs), instead of IE-72 specific T cell clones, in order to show that the *in vivo* HCMV-specific host immune response is also able to kill Titan-ΔUS28-infected monocytes; we saw reductions in the levels of virus reactivation events from monocytes infected with Titan-ΔUS28 similar to the levels seen with those infected with Titan-WT after their subsequent differentiation and maturation (Fig. 10C).

To confirm that these observations were a result of T cell killing rather than, for instance, repression of GFP expression, we also incubated monocytes infected with Titan-WT or Titan-ΔUS28 with isolated CD8^+^ T cells from matched donors and analyzed T cell-specific increases in the levels of two markers of T cell activation, CD69 and 4-1BB, by flow cytometry to assay for CMV-specific CD8^+^ T cell responses. The CD8^+^ T cells exposed to Titan-ΔUS28-infected monocytes were more highly activated than those exposed to monocytes latently infected with Titan-WT (Fig. 10D). As a readout of the functional capacity of these CMV-specific CD8^+^ T cells, we also analyzed their production of the cytokines gamma interferon (IFN-γ) and tumor necrosis factor alpha (TNF-α) as well as upregulation of degranulation marker CD107a and expression of granzymes A, B, and K. Figure 10E shows that, by the use of these functional effector markers, CD8^+^ T cells clearly recognized Titan-ΔUS28-infected monocytes but not Titan-WT-infected monocytes. We also confirmed that monocytes latently infected with Titan-WT virus can act as targets for CTLs; the levels of expression of CD107a as well as the levels of production of granzymes, IFN-γ, and TNF-α by CMV-specific CD8^+^ T cells in response to the Titan-ΔUS28 virus were equivalent to the levels seen in response to monocytes which had been infected with Titan-WT virus and pulsed with IE1/2 peptides as positive-control targets for CMV-specific CD8^+^ T cell responses. These data, taken together, argue that monocytes infected with Titan-ΔUS28 are robustly detected by preexisting CD8^+^ T cells in HCMV-seropositive donors.

Finally, we tested whether treatment of latently infected cells with VUF2274 also made them targets for IE72-specific CTLs. Figure 10F shows that, consistent with previous analyses (37, 73, 81), monocytes latently infected with simian virus 40-GFP-TB40E (SV40-GFP-TB40E) were detectable as GFP^+^ cells and that their numbers remained relatively constant when cultured with IE72-specific CTLs. In contrast, when these latently infected monocytes (GFP^+^ cells) were treated with VUF2274, a steady reduction of the number of latently infected cells was observed in these IE72-specific CTL cocultures (Fig. 10F). Unfortunately, due to the long-term toxicity of VUF2274, we were not able to show that this also resulted in a subsequent reduction in reactivation events after monocyte differentiation and maturation to mDCs.

Taken together, these data suggest that the preexisting CTL response to HCMV, in healthy carriers, is able to target and kill monocytes infected with Titan-ΔUS28 and that the treatment of latently infected monocytes with US28 inhibitor VUF2274 also makes them novel CTL targets. On this basis, we suggest that inhibition of US28 with, for instance, small-molecule inhibitors could result in untimely reactivation of latent virus and could allow their targeting by preexisting HCMV-specific host T cell responses.

## DISCUSSION

HCMV latency and reactivation of virus from latency pose significant clinical threats to immunosuppressed transplant recipients and other immunocompromised individuals (82). However, there are currently only a few published strategies for treatment of HCMV latency (17, 40, 73). HCMV establishes latent infection in early myeloid lineage cells (4), where its latent life cycle is characterized by expression of only a small subset of viral genes independently of viral IE gene expression. This includes expression of the viral chemokine receptor homologue US28 (35, 83). US28 expression during HCMV lytic infection is well established to activate multiple cell signaling pathways which can activate the viral MIEP (48, 52–57). These signals have previously been linked to vascular diseases as well as to oncomodulation (53, 65, 66). However, this powerful signal activation appears to contrast with the recently identified requirement for US28 expression to establish a latent infection in CD34^+^ stem cells, likely by repressing viral IE gene expression (21).

Here, we confirm this important function of US28 during latent infection in CD14^+^ monocytes and, in part, solve this paradox by showing that US28 appears to have very different effects on cell signaling in undifferentiated and differentiated cells. US28 attenuates cellular signaling of MAP kinase and NF-κB in undifferentiated cells, which supports epigenetic suppression of the MIEP to prevent lytic infection. In contrast, US28 activates these same signaling pathways in differentiated myeloid cells to help drive IE expression and virus reactivation. Our analysis, using a viral isolate with a deletion for the *US28* gene, showed that US28 was necessary for the maintenance of HCMV latency in monocytes; monocytes infected with Titan-ΔUS28 underwent full lytic infection and produced infectious virus.

These initial analyses were made 7 days postinfection. Interestingly, repeating this analysis at early times postinfection indicated that this ability of Titan-ΔUS28 to initiate a lytic infection in monocytes was immediate and did not require time for monocytes to become differentiated to a macrophage/DC phenotype, arguing that US28 does not function by suppressing myeloid differentiation and subsequent viral reactivation. We observed IE mRNA expression at 12 h postinfection, IE protein expression at 24 h postinfection, and UL32-GFP expression at 48 h postinfection, indicating that infection of monocytes by Titan-ΔUS28 undergoes a time course of gene expression similar to that seen with lytic infection of fibroblasts. Additionally, we did not see changes in cell surface markers of myeloid differentiation 7 days postinfection with Titan-ΔUS28. This suggests that, in the absence of US28 protein, monocytes themselves can support lytic HCMV infection and that differentiation of monocytes is not necessary for lytic infection under these conditions.

Complementation analyses in THP-1 cells stably expressing US28 functional mutations gave us substantial insight into the mechanisms by which US28 suppressed lytic infection of monocytes. Infection with Titan-ΔUS28 of THP-1 cell lines stably expressing different US28 mutants, including wild-type US28 protein (HA-US28-WT), US28 protein which cannot signal (HA-US28-R129A), and US28 protein which cannot bind chemokines (HA-US28-Y16F), showed that US28 maintains latency by G protein-mediated signaling and that this signaling occurs in a constitutive manner, independently of chemokine binding. These observations fitted well with analyses of the effect of US28 on the MIEP in transfection assays. Nucleofection of our HA-US28 constructs into THP-1 cells which had been transduced by lentivirus to express eGFP driven by the MIE promoter confirmed that HA-US28-WT repressed the MIEP in a signaling-dependent manner and further showed that, in differentiated THP-1 cells, the activity of US28 switched from that of a repressor, as seen in undifferentiated monocytic cells, to that of an activator of the MIEP and that this, too, was dependent on the US28 signaling capacity. This was entirely consistent with data from other permissive cell types which have consistently shown the ability of US28 to activate IE expression (63, 64). Our view, that the regulation of IE expression by US28 was likely signaling dependent, was confirmed by our observations indicating that US28 profoundly affected the level of a number of cellular phosphokinases. In undifferentiated monocytic cells, HA-US28-WT significantly attenuated the MAP kinase pathway; in particular, ERK1/2, MSK-1, and CREB were all less phosphorylated. Similarly, consistent with the observation that US28 has an activation effect on the MIEP in cells that are permissive for HCMV lytic infection (52, 63, 64), US28 activated MAP kinase signaling in differentiated monocytic cells and this is diametrically opposite its suppressive effect in undifferentiated monocytic cells. US28 also differentially affected NF-κB localization in undifferentiated and differentiated monocytic cells; US28 resulted in increased cytoplasmic NF-κB localization in undifferentiated cells but enhanced NF-κB nuclear localization in differentiated cells. This, again, is consistent with the known NF-κB-mediated activation of the MIEP (63) and helps explain the differentiation-dependent reversal of US28 activity with respect to the chromatin-mediated control of MIEP activity and IE gene expression in undifferentiated or differentiated monocytic cells, which suppress or support IE gene expression, respectively. Taking the data together, it appears that US28 activity in early myeloid lineage cells serves to maintain latency by attenuating reactivation signals, such as MAP kinase (23) and NF-κB, which are both known to activate the MIEP either directly or via release of chromatin-mediated suppression around the MIEP (23, 47, 63, 84, 85). We were able to observe this change in activation and repressive chromatin marks on the MIEP by ChIP assay at the level of H3 phosphorylation and HP1 recruitment. This radical differentiation-dependent reversal of US28 activity helps resolve the problem of why US28, considered to be a strong activator of cellular signaling in lytic infection, is also expressed during latency, when lytic infection is known to be actively suppressed.

How US28 apparently reverses its signaling properties between undifferentiated and differentiated myeloid cells in such a significant manner remains unclear. US28 has been investigated in a range of different cell types, but its effects on different cell types have not always been consistent (56, 65). Our observations appear to represent the first evidence that US28 can attenuate cell signaling, independently from other viral GPCRs, in a constitutive manner, at least in undifferentiated myeloid cells. Two models may explain these observations. First, phosphorylation of the C-terminal tail of US28 is known to modify its signaling properties (70–72), and US28 is known to be phosphorylated by protein kinase C (PKC) (71), showing isoform changes during myeloid cell differentiation (86). It is, therefore, possible that changes in cellular kinase expression that occur during myeloid differentiation alter US28 signaling by its differentiation-dependent phosphorylation. Alternatively, US28 is known to interact promiscuously with a range of different G-alpha proteins (47, 49, 50); similarly, changes in cellular G-alpha protein expression are known to occur during myeloid differentiation. Consequently, differentiation-specific changes in the G-alpha protein interactions with US28 could lead to changes in the signaling properties of US28.

More recently, US28 has been shown to activate PLC-β in monocytes (58), via G protein-coupling, in a chemokine-independent manner, which suggests that attenuation of the MAP kinase and NF-κB signaling pathways may not be the only mechanism by which US28 may affect IE expression differently in undifferentiated and differentiated monocytic cells. Given that histone deacetylase (HDAC) inhibitors induce IE gene expression in otherwise latently infected monocytes but do not trigger full, lytic gene expression or virus production in treated monocytes (17), it appears that major IE protein expression alone in monocytes is not sufficient to induce full virus production. Since deletion of the US28 gene causes full lytic gene expression, US28 is likely to have functions beyond just repression of the MIEP.

Our attempts to reproduce US28-mediated attenuation of the lytic cycle in undifferentiated cells, using small-molecule inhibitors of either MAP kinase or NF-κB in isolation, had only a limited effect on the ability of Titan-ΔUS28 to establish lytic infection of monocytes. However, concomitant inhibition of MAP kinase and NF-κB signaling pathways profoundly reduced lytic gene expression in Titan-ΔUS28-infected monocytes. Our view is that lytic infection, via activation of the MIEP, can be stimulated by either MAP kinase or NF-κB signaling but requires activation of at least one of these two pathways. Our observation that delaying this treatment of inhibitors until 24 h postinfection no longer prevented lytic infection demonstrates that US28 has to act at a very early time point postinfection to suppress activation signals to the MIEP and thus prevent lytic infection. We believe that these activation signals are likely triggered by viral binding or entry into the cell, perhaps triggering innate immune responses, which could lead to the activation of the MIEP (87, 88), and that US28 may serve to attenuate this response and thereby stifle IE activation to help initiate viral latency.

Finally, on the basis of our findings that US28 is crucial to establish HCMV latency in monocytes by suppressing IE gene expression and subsequent lytic infection, we predicted that inhibition of US28 activity, using its inverse agonist VUF2274, would stimulate lytic gene expression in normally latently infected monocytes. This was indeed the case and led to a proof of principle that inducing lytic infection in monocytes could lead to their targeting by preexisting host HCMV-specific CTL responses. First, IE72-specific CD8^+^ T cell clones reduced viral reactivation from monocytes infected with Titan-ΔUS28 compared to monocytes infected with Titan-WT virus; second, similarly, treatment of experimentally latent monocytes with VUF2274 also made latently infected cells targetable by these IE72-specific CD8^+^ T cell clones. We also demonstrated that monocytes infected with Titan-ΔUS28 are targets for PBMCs from healthy HCMV-positive donors and confirmed that this was mediated by classical CTL killing on the basis of staining for markers of T cell activation and degranulation.

Although this approach of “shock and kill,” using HDAC inhibitors, has already been demonstrated to be effective against latent HCMV (17), HDACs have a wide range of biological functions and the inhibition of HDACs could have significant off-target effects. Consequently, an inhibitor of US28 usable for “reactivation” of IE expression could be an attractive alternative, particularly in healthy seropositive tissue donors where reactivation events are thought to be subclinical (89, 90). We do note that VUF2274 did show some cytotoxicity, likely due to off-target effects, possibly including the inhibition of CCR1. However, the structure of the US28 protein has recently been solved (46) and could aid the development of more-specific small-molecule inverse agonists of US28.

Taken together, our observations point to a crucial role for viral US28 in the establishment of HCMV latency in monocytes which is mediated by differentiation-dependent US28 signaling and indicate that inhibition of US28, resulting in the induction of IE expression in normally latently infected cells, could aid in novel immunotherapeutic strategies to target and clear the HCMV latent reservoir in certain clinical settings.

## MATERIALS AND METHODS

### Cell culture and virus infection.

Viral isolates of the Titan wild type (Titan-WT) and the equivalent isolate with a deletion in the US28 gene (Titan-ΔUS28) which have a UL32-GFP tag have been described previously (68), as has an isolate of TB40/E, a wild-type clinical isolate of HCMV carrying an SV40-GFP expression cassette tag (termed "SV40-GFP-TB40E") which allows the detection of latently infected cells (81), as well as an isolate of TB40/E with an IE2-YFP tag (termed "RV1164") (40). Primary CD14^+^ monocytes were isolated from apheresis cones (NHS Blood and Transfusion Service, United Kingdom) as described previously (90) and cultured in X-vivo15 (Lonza) at 37°C in 5% CO_2_. Cells were infected with all HCMV viral isolates at a predicted multiplicity of infection (MOI) of 5 (based on infection of RPE-1 cells), leading to 10% latently infected cells as determined by GFP expression upon infection with SV40-GFP-TB40E. Monocytes were activated to differentiate into immature dendritic cells by granulocyte-macrophage colony-stimulating factor (GM-CSF) and interleukin-4 (IL-4) (PeproTech) stimulation at 1,000 U/ml for 5 days. Mature dendritic cells were produced by stimulation for 2 further days with lipopolysaccharide at 500 ng/ml.

THP-1 cells were infected for 3 days with Titan-WT, Titan-ΔUS28, or SV40-GFP-TB40E at a predicted MOI of 5 (based on infection of RPE-1 cells) and were differentiated using 50 ng/μl phorbol myristate acetate (PMA) as previously described (91). To quantify reactivation/virus release, cells were cocultured with 3 × 10^3^ fibroblast cells per cm^2^ of growth area in a 50:50 mixture of Dulbecco's modified Eagle's medium 10 (DMEM-10) and RPMI-20 or X-vivo15 (Lonza).

### RT-qPCR.

Monocytes were infected with Titan-WT or Titan-ΔUS28. After 3 h of incubation, cultures were washed with citrate to remove cell-associated virus. Samples for each condition were then harvested immediately into TRIzol (Life Technologies, Inc.) to act as control wells for input mRNA from incoming virions. Other cultures were incubated for the duration of time indicated for each experiment (between 1 and 7 days) before also being harvested in TRIzol, and mRNA was then isolated using a miRNeasy minikit (Qiagen), following the manufacturer’s instructions. UL138, IE, and UL99 were quantified using one-step reverse transcription-qPCR (RT-qPCR) and a QuantiTect virus kit (Qiagen) as previously described (41). Values were calculated using the threshold cycle (ΔΔ*C*_*T*_) method and expressed as relative changes between the input control and the relevant time point. Samples were normalized to GAPDH (glyceraldehyde-3-phosphate dehydrogenase), as described previously (41).

### IF staining and microscopy.

Cells were fixed and stained as described previously (92). In brief, following fixation in paraformaldehyde and permeabilization in 70% ethanol, cells were washed in phosphate-buffered saline (PBS) and stained with mouse anti-IE (Argene 11-003) diluted at 1 in 1,000 in PBS containing 10% goat serum. This was followed by detection with goat anti-mouse antibody (Alexa Fluor 594). Where indicated, goat anti-GFP (fluorescein isothiocyanate [FITC] conjugated; Abcam, Inc.) and Hoechst 33258 nuclear stain were added simultaneously.

### Virus release assays.

Media was harvested from infected monocyte cultures and titrated onto human foreskin fibroblasts (HFFs). Following 3 h of incubation with rocking at room temperature, the cultures were then washed, media were replaced, and the cultures were incubated for a further 24 h at 37°C in 5% CO_2_. Fibroblasts were then stained for immediate early antigen, using the protocol described above, and the virus titer was calculated as the number of infected cells per microliter of monocyte supernatant media added to the fibroblast culture.

### Lentiviral transduction of US28 expression and confirmation of expression by Western blotting.

Lentiviral US28 expression constructs where US28 expression is driven by a spleen focus-forming virus (SFFV) promoter with an N-terminal hemagglutinin tag (HA) were kindly provided by Daniel Streblow (Oregan Health and Science University) and transduced into THP-1 cells, followed by confirmation by Western blotting, as previously described (73).

### Detection of phosphoproteins by immunoblotting.

THP-1 cells, transduced to express various constructs of HA-US28, were lysed in radioimmunoprecipitation assay (RIPA) buffer, and nuclei and cell debris were removed by centrifugation at 13,000 × *g* for 10 min at 4°C. Proteins were separated on 10% SDS-polyacrylamide gels and transferred to nitrocellulose membranes (Axygen; Corning). Incubations with primary and secondary antibodies were performed using 5% skimmed milk for 1 h each at room temperature. Proteins were detected using the following antibodies: anti-p42/p44 or phosphor-anti-p42/p44 antibodies or anti-MSK1 or anti-phosphor-MSK1 antibodies (serine 360) (all 1:1,000; Cell Signalling Technology, Danvers, MA) or anti-CREB or phosphor-CREB antibodies (S360) (both Merck). The secondary antibody used was chicken anti-rabbit horseradish peroxidase (Santa Cruz Biotech). Blots were developed with the use of enhanced chemiluminescence (GE Healthcare) and visualized with autoradiography film.

To detect cellular localization of NF-kB, cells were fractionated using REAP (rapid, efficient, and practical) (93) and proteins detected using the following antibodies: anti-NF-κB (Abcam, Inc.), anti-p84 (Thermo), and anti-GAPDH (Millipore). Secondary antibodies used were chicken anti-rabbit and bovine anti-mouse horseradish peroxidase (both Santa Cruz Biotech).

### MIEP activation/repression assays using THP-1 cells.

THP-1 cells expressing an MIEP-eGFP construct have been previously described (76). These cells were transfected by nucleofection with various US28 constructs, using Amaxa Cell Line Nucleofector Kit R (Lonza); MIEP-driven eGFP expression was detected and measured using a BD Accuri C6 flow cytometer, where dead cells were excluded from analysis by staining with Zombie Red fixable viability dye (BioLegend); and mean fluorescence intensities for eGFP expression were analyzed within the same experiment using FlowJo software (Tree Star Inc.).

### Phosphokinase arrays.

For phosphokinase antibody arrays, THP-1 cells were transduced with lentiviral US28 expression vectors, as described previously (73). Cells were harvested and lysed following the manufacturer’s protocol (Proteome Profiler human phospho-kinase array kit; R&D Systems), and spot intensity was analyzed using ImageJ software.

### IE-specific T cell and PBMC killing assays.

CD14^+^ monocytes were plated at 1 × 10^5^ cells per well of a 96-well plate and infected with Titan-WT, Titan-ΔUS28, or SV40-GFP-TB40E. Three days postinfection, the monocytes were then cocultured with HLA-matched (HLA-A2) immediate-early-specific CD8^+^ T cells (94) at an effector-to-target cell (E:T) ratio of 5:1 or with donor PBMCs at an E:T ratio of 1:3. VUF2274 was added to the relevant wells at a concentration of 6 × 10^−7^ M. In order to measure the killing of latently infected monocytes, the numbers of HCMV-infected, GFP-expressing, or UL32-GFP-expressing monocytes were enumerated, by fluorescence microscopy, over several days as indicated. Media from these cultures were collected to determine the titer of any virus that was produced by Titan-ΔUS28-infected monocytes before they were differentiated to dendritic cells by cytokine treatment. To quantify virus reactivation events, these monocyte-derived dendritic cells were cocultured with indicator fibroblasts for 2 weeks and reactivation events enumerated by IF staining for IE and by counting IE-positive foci.

### Flow cytometry.

Experimentally infected monocytes were coincubated with CD8^+^ T cells from the same, seropositive donor overnight in the presence of CD107a Alexa Fluor 647, 5 μg/ml brefeldin A, and 2 μM monensin (all from BioLegend) at 37°C. CD8^+^ T cells were harvested and washed and then stained with a combination of surface antibodies (CD3 brilliant violet 650, CD14 brilliant violet 510, and CD19 brilliant violet 510; BioLegend) and LIVE/DEAD Fixable Aqua Dead cell stain (Invitrogen) at 4°C. Cells were fixed and permeabilized using FIX & PERM (ADG, Kaumberg, Austria) and stained intracellularly with antibodies (CD69 Pacific Blue, 4-1BB phycoerythrin [PE]-Cy5, CD8 brilliant violet 570, and granzyme A FITC [BioLegend]; granzyme B FITC [Miltenyi Biotec, Inc.]; granzyme K FITC [Santa Cruz Biotechnology, TX, USA]; TNF-α brilliant ultraviolet 395 and IFN-γ brilliant violet 786). Responding CD8^+^ T cell populations were identified by the expression of CD69 and 4-1BB at levels above the background, and expression levels of CD107a, TNF-α, and IFN-γ were then measured. In all cases, cell doublets, monocytes, B cells, and dead cells were eliminated from the analyzed populations.

To analyze differentiation markers, monocytes were labeled with anti-CD14 and anti-CD83 antibodies (BioLegend) (both allophycocyanin [APC] conjugated).

### Chromatin immunoprecipitation.

Chromatin immunoprecipitation of the MIEP was performed using a Sigma Imprint ChIP kit and antibodies against S10P and HP1 (HP1 antibody [FL-191; catalog no. sc-28735] and anti-ser-10-H3 [phospho-histone H3 {Ser10} antibody {9H12L10}]; ABfinity Rabbit Monoclonal). The MIEP was quantitated against a standard curve of viral DNA, analyzed by qPCR, and then plotted as a percentage of input DNA, with each sample run in triplicate. The primers and probe used were as follows: forward primer, CCAAGTCTCCACCCCATTGAC; reverse primer, GACATTTTGGAAAGTCCCGTTG; probe, 6-carboxyfluorescein (FAM)-TGGGAGTTTGTTTTGGCACCAAA-6-carboxytetramethylrhodamine (TAMRA).

### Ethics statement.

All human samples were obtained under ethical approval by and after approval of protocols from the Cambridgeshire 2 Research Ethics Committee (REC reference 97/092) conducted in accordance with the Declaration of Helsinki. Informed written consent was obtained from all of the volunteers included in this study before providing blood samples, and all experiments were carried out in accordance with the approved guidelines.
