# Supplementary figures and images for: Latency-Associated Expression of Human Cytomegalovirus US28 Attenuates Cell Signaling Pathways To Maintain Latent Infection
Source: mBio. 2017 Dec 5;8(6):e01754-17. doi: 10.1128/mBio.01754-17 (PMC5717388; doi:10.1128/mBio.01754-17)

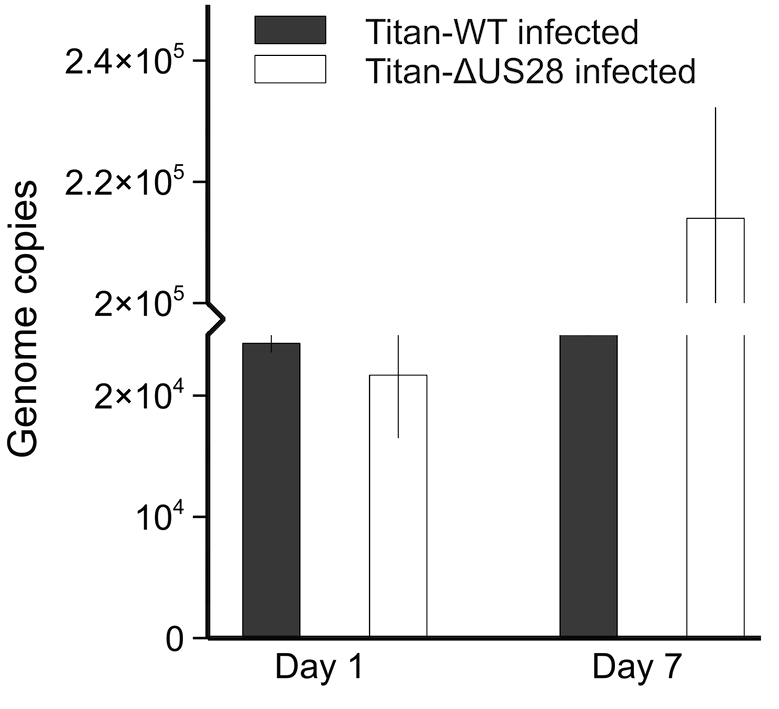

Supplement: FIG S1 [file mbo006173617sf1.tif]

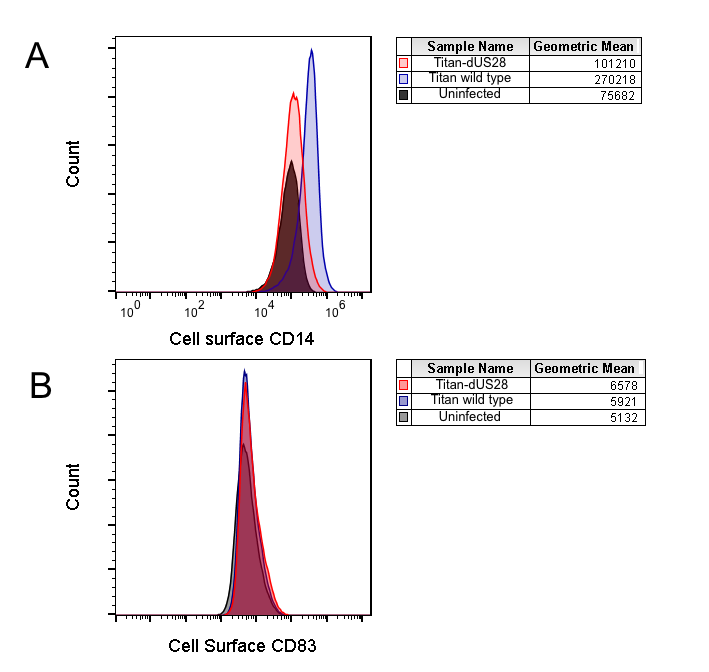

Supplement: FIG S2 [file mbo006173617sf2.tif]

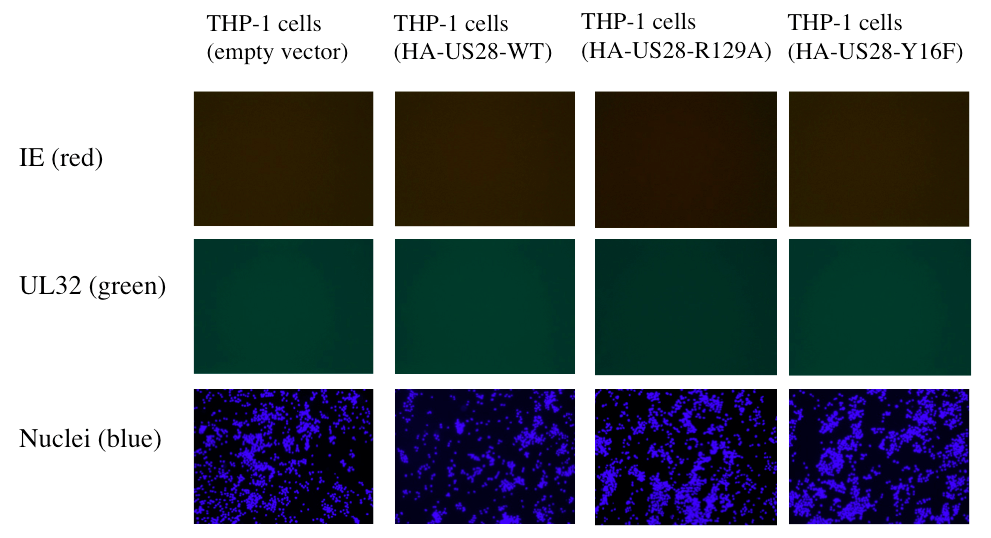

Supplement: FIG S3 [file mbo006173617sf3.tif]

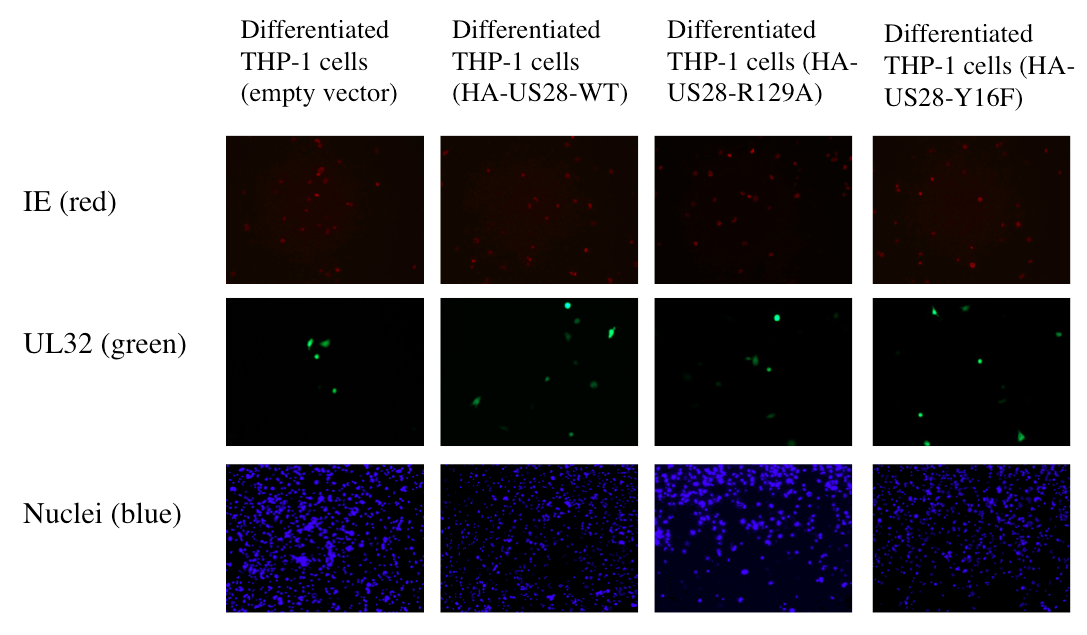

Supplement: FIG S4 [file mbo006173617sf4.tif]

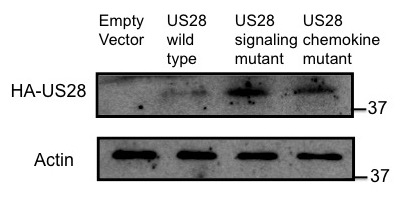

Supplement: FIG S5 [file mbo006173617sf5.tif]

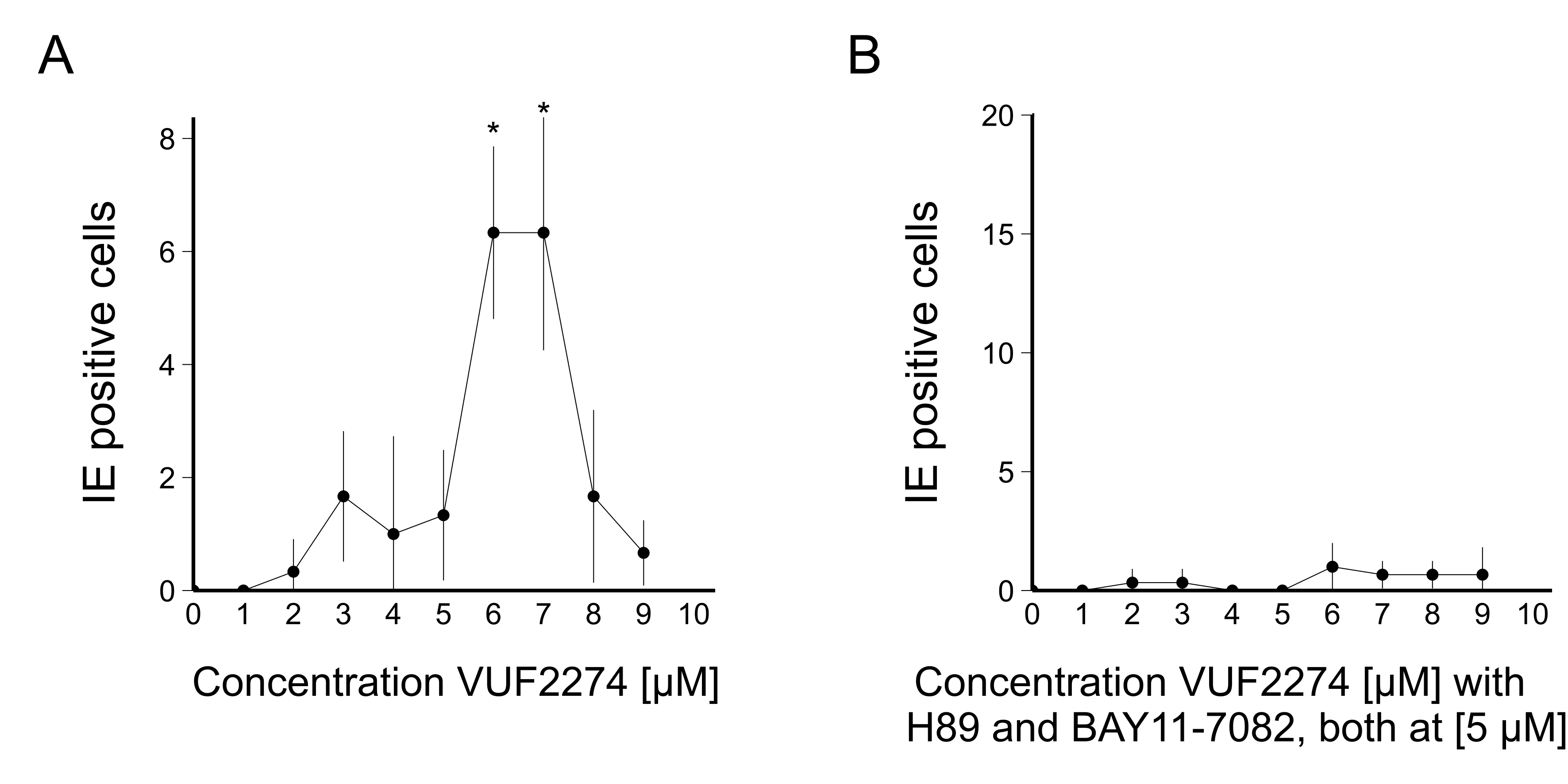

Supplement: FIG S6 [file mbo006173617sf6.tif]
